# Proposal of a Nosological Classification of the Diseases of Children

**Published:** 1820-12

**Authors:** A. B. Granville


FOR THE LONDON MEDICAL AND PHYSICAL JOURNAL.

### Proposal of a Nosological Classification of the Diseases of Children.

By A. B. Granville, M.D. F.R.S. &c.

HAVING, in my capacity of principal physician to the Royal Infirmary for Sick Children, deemed it necessary to draw up the accompanying synoptical arrangement of the various morbid affections to which infancy is known to be most liable, I beg to be allowed to give it, through the medium of your Journal, a more general circulation; that, if it be found useful, it may have the chance of being more generally adopted; or, if considered defective in any way, I may reap the benefit of whatever corrections its publication may suggest.

You must have been struck, in common with every other practitioner of this or any other country, with the singular circumstance of no regular classification of infantile diseases having ever been proposed by the several eminent authors who have written on that particular branch of medicine; and you will probably agree with me, that no where is the necessity of such a classification likely to be more felt than in an extensive institution directed solely to the alleviation of those diseases, where daily and numerous occasions must occur for its use.

In the Infirmary to which I have had the honour of being appointed principal medical officer, it would scarcely have been possible for the physicians and surgeons belonging to it, (considering the number of stations at which the sick children of the poor are relieved, without any distinction, or the imposition of any of the usual shackles attendant on public charities,) to have preserved any degree of uniformity in the medical records of that Institution, had we relied on the usual routine only, of entering the names of patients without any very precise and uniform designation of their complaints. It became, therefore, necessary to establish something like a *synopsis*, by the help of which, as if with the use of a common language, we might become intelligible, not only to each other, but also to the profession in general; the members of which may hereafter wish to refer, for many useful subjects of information, to the registers of the Royal Infirmary.

How far the present attempt to form such a synopsis may succeed in removing the difficulties which, like those experienced in general practice before any nosological arrangement of diseases had been established, must be felt in the consideration of infantile complaints, I leave my colleagues and the public to decide. I have no pretensions on the subject, and I give my present speculation as a mere attempt; happy if it serve to stimulate more competent persons to fill up the chasm which has hitherto existed in nosological science. The names of Heberden, Rosen, Capuron, Burns, and Underwood, not to mention several others, must be familiar to those who have paid particular attention to the diseases of children. The descriptive observations and practical remarks of these eminent men leave scarcely any thing to be wished for; yet it must be admitted that, had those descriptions and remarks followed some specific synoptical arrangement, the facilities for their study and their retention would have been greater than they now are. A classification in any science is but a sort of artificial memory, by means of which our intellect can travel through the otherwise perplexing mazes of that science with comparative facility. A classification teaches no science, but materially assists us in the study of it. Who is then prepared to say that botany, mineralogy, and even medicine, have not made greater progress since the adoption of a synoptical arrangement of the objects which constitute their study, than they could be expected to make without any such arrangement? I am aware that a very eminent botanist, "yet young in age, though old in fame,"* has said, that nature designed no classes of plants, no orders, nor even genera, but only created species: but is it not to facilitate the study of the latter so bountifully strewed on the earth's surface, that the former artificial divisions have been established? and may we not assert with confidence, that, but for such artificial divisions in the natural sciences, any tolerable degree of proficiency in them would require a much longer period of study than it does at present? Surely it is not in our days that we are to be told that a botanist could, with equal ease to himself, have studied, named, and described a new plant, before the adoption of the classifications proposed by Tournefort, Linnæus, or Jussieu, as he can now; or that a mineralogist could satisfy himself respecting the nature of an undescribed mineral, without some one of the methodical arrangements which have been published within the last half century. These observations may in the like manner be applied to medicine, which, but for the different systems of nosology, would at this moment offer a mere mass of facts, difficult to be retained; more difficult to be deciphered; and still more difficult to be applied to practical purposes, except, perhaps, on a few occasions of empirical investigation.

Thus far for the necessity as well as the propriety of classifications. With regard to that which I venture to propose for infantile complaints, I may remark, that I have taken a few hints in its formation from one or two of the writers I have had occasion already to mention, and that I have entirely departed

* Mr. Robert Browne.

from the artificial division of those diseases which I proposed more than twelve months ago, in my *Reports on the Practice of Midwifery at the Westminster General Dispensary.* The plan of the present classification is as follows:

I have, in the first place, considered all deviations from healthy action or healthy structure in children, as having either begun with their fetal life and formation, or developed themselves at the moment, and in consequence of, parturition. These therefore form a first great division, very distinct from that of any other complaint which may afflict a child subsequently to its birth and up to the adult age.

To this first great division I have applied the denomination of "Morbi Congeniti," and I have necessarily been obliged to make two classes of it; in the former of which I have placed all "diseases or deviations from healthy structure previous to, and consequently independent of, parturition;" while in the second "those congenite diseases have been arranged which are connected with, or dependent on, parturition." The Greek word ATOCICA* sufficiently designates, in my opinion, the generality of the former diseases; while the word TOCICA† not unaptly applies itself to those of the second class.

The diseases embraced by the first of these two classes of the first great division are by far more numerous than those of the second class; and, in order to facilitate their recollection, they have been subdivided into ten orders, each order containing an unequal number of genera. I have not extended my consideration to either species or varieties, as this would have carried me too far. The appellations to be given to the ten orders was not a matter of indifference; but a little reflection on the particular parts of the fetal structure or functions which are known, from the accumulated observations of authors and from daily occurrence, to be mostly affected, soon presented me with what will be considered appropriate designations. Thus, in congenite deviations from healthy action or structure, we remark that they consist in, 1, natural openings imperforate; 2, unnatural adhesions; 3, unnatural separations; 4, deficient organization; 5, superfluous organization; 6, unnatural accumulation of fluids; 7, displacements; 8, conformations wanting symmetry; 9, defective action of the senses; and lastly, 10, marks. These would, therefore, constitute more naturally the orders. To express in a single word, for the sake of brevity and clearness, the sense implied by the above paraphrastic expressions, the Greek language has been adopted,‡ in imitation

* From α privative, and τοκ'ος *partus.*

† From τοκ'ος, *partus.*

‡ 1, ATRESIS, from ατρητ'ος, *imperforatus;* 2, COLLISIS, from κόλλάω, *glutino;* 3, DIAZUXIS, from διαζευξης, *separo;* 4, ELATTOSIS, from ἐλλιπὴς, *imperfectus,*

of the best masters in nosology; but I have been sparing of that language in naming the different genera, the most familiar Latin denominations of which have been preferred, affixing to them a collateral English translation or nomenclature. The first class embraces forty-six genera.

Two orders only are contained in the second class: 1, *Topical;* 2, *universal;* each being subdivided into five genera.

Having thus disposed of all the diseases which, from their being coeval with the patient, form a well-defined and distinct division, it remained for me to arrange all those deviations from health which are known to occur subsequently to birth and as late as the adult age, whether peculiar to the period of time that lies between those two epochs, or likely to affect children in common with persons more advanced in years. These two considerations gave rise to a second great division, which I have designated by the name of "Morbi Subsequentes," as quite distinct from those which formed the subject of the preceding remarks.

In the subdivision of this second great section, some difficulties occurred. After some ineffectual attempts, I determined to adopt the most modern physiological divisions of the animal functions as the basis of my classification:* but, as nosology should always be directed to facilitate, rather than obstruct by a multiplication of difficulties, the study of diseases, it occurred to me that, if I adopted each function as a class, the number of classes in the second division would have been four times greater than that in the first division, and consequently more difficult to be retained. I have therefore contrived to group these functions in such a manner as, I trust, that, with a very little stretch of literal signification, the groups may serve as classes; while the functions themselves would naturally fall in as orders. Hence, by considering *respiration* and *circulation* as the two functions which eminently tend to preserve life, a class composed of two orders, 1, PNEUMATICI,† 2, HÆMATICI,‡ was formed, to which the Greek word ZOTICA§ was applied, and in which the "morbid alterations of the preservative functions" were arranged." Proceeding by the same rules, *digestion* and

---

*cui deest aliquid*, which ought perhaps to have been adopted in preference to the former; 5, PERISSEUZIS, *redundans;* 6, HYDROPS, from ὕδωρ, *aqua;* 7, ECTOPIÆ, from ἔκτοπος, *è loco suo emotus;* 8, ASYMMETRIA, from α priv. and συμμετρία, *symmetria;* 9, PARÆSTHESIS, from παραίσθησις, *sensus imperfectus;* 10, METROCELIS, from μήτηρ, *mater*, and κηλὶς, *macula*.

* These functions are, 1, respiration; 2, circulation; 3, digestion; 4, absorption; 5, secretion and excretion; 6, exhalation; 7, sensation; 8, voluntary motion; (9, generation,)

‡ From αἷμα, *sanguis*.

*absorption* being looked upon as functions tending, particularly in children, to the increase of the structural mass, a second class was established composed also of two orders, 1, Cœliaci,* 2, Lymphatici,† which, under the Greek appellation of Auxitica,‡ admitted the arrangement of all the "morbid alterations of the augmentive functions." A third class was formed of the functions *secretion*, *excretion*, and *exhalation*, which gave me an opportunity of adopting the two orders, 1, Eccritici,§ 2, Dermatici,‖ and of grouping them under the title of Apocritica,¶ containing the "morbid alterations" of those particular functions, which, from their ultimate office of separating and purifying, I have called the "segregating functions." To the fourth and last class, the Greek word Œsthetica,** embracing the "morbid alterations of the sensitive functions," has been applied, and is composed, like the three preceding classes, of two orders, 1, Neurotici,†† or diseases affecting sensation; 2, Myotici,‡‡ or diseases affecting voluntary motion. The first class contains twenty-four, the second class seventeen, the third class thirty, and the fourth class seventeen, genera. The function of generation, for obvious reasons, has not been, nor could it be, taken into consideration, when treating of infantile diseases.

Having given an explanation of the motives which induced me to undertake this trifling labour, and of the mode in which it has been executed, I proceed to give the Synopsis itself, in the drawing-up of which I freely acknowledge that I have derived the assistance of two or three Greek names adopted from Dr. Young and Dr. Mason Good. Dr. Macbride, who formed of infantile complaints a fourth class of his General Nosology, has been of no service to me; nor has any other author besides who has written specifically on the nature and treatment of complaints in children.

The Synopsis I thus submit to the public through your Journal, has been printed and published within these few days, on a moderate-sized card, for the convenience of students.

---

† From νυμφη, *quasi aqua.*

‖ From δέρμα, *pellis,* the organ of exhalation.

¶ From αποκρινω, *segrego.*

** From αισθάνομαι, *sentio, sensû corporis.*

†† From νευρον, *nervus.*

‡‡ From μυων, *musculus.*

*Synopsis Nosologica Morborum, quibus Infantes ac Pueri tentantur, Legibus Physiologicis, recensita; et in Usu Nosocomii Regalis ad Morbos præsertim Puerorum debellandos*, ab AUGUSTO B. GRANVILLE, M.D. S.R.S. *statuta.*

### Divisio Prima.

### MORBI CONGENITI.

### CLASSIS I. ATOCICA.—*Congenite Diseases, independent of Parturition.*

#### Ordo I. ATRESIS.—Natural openings imperforate.

| | | |
|---|---|---|
| G. 1. | Pupillæ occlusio | *Closed pupil* |
| 2. | Aurium occlusio | *Closed ears* |
| 3. | Narium coalitus | *Closed nostrils* |
| 4. | Anus occlusus | *Imperforate anus* |
| 5. | Præputium occlusum | *Imperforate præputium* |
| 6. | Urethra occlusa | *Imperforate urethra* |
| 7. | Vagina imperforata | *Imperforate vagina* |
| 8. | Uterus imperforatus | *Imperforate womb* |

#### Ordo II. COLLISIS.—Unnatural adhesions.

| | | |
|---|---|---|
| 9. | Palpebrarum conglutinatio | *Adhesion of the eye-lids* |
| 10. | Linguæ cum gingivis concretio | *Adhesion of the tongue to the gums* |
| 11. | Lingua frænata | *Tongue-tied* |
| 12. | Digitorum coalitus | *Union of two or more fingers or toes* |
| 13. | Penis cum scroto agglutinatio | *Adhesion of the penis to the scrotum* |

#### Ordo III. DIAZUXIS.—Unnatural separations.

| | | |
|---|---|---|
| 14. | Labium Leporinum | *Hare-lip* |
| 15. | Lagostomatum palatinum | *Divided palate* |
| 16. | Rima in perinæo | *Slit perineum* |
| 17. | Hypo et Epispadias | *Irregular openings of the urethra* |

#### Ordo IV, ELATTOSIS.—Defective organization.

| | | |
|---|---|---|
| 18. | Monoculus | *Having only one eye* |
| 19. | Monotestis | *Having only one testicle* |
| 20. | Digitorum defectus | *Deficient number of fingers or toes* |

#### Ordo V. PERISSEUZIS.—Superfluous organization.

| | | |
|---|---|---|
| 21. | Digiti superflui | *Additional fingers or toes* |
| 22. | Excrescentiæ | *Excrescences* |

#### Ordo VI. HYDROPS.—Unnatural accumulation of fluids.

| | | |
|---|---|---|
| 23. | Hydrencephalus | *Water in the brain* |
| 24. | Hydrorachitis | *Watery tumors of the spine* |
| 25. | Hydrocele | *Fluid tumor of the testicle* |
| 26. | Hydrogenesis | *Infiltration of external parts of generation* |
| 27. | Mammarum inflatio | *Swelling of the breasts* |

#### Ordo VII. ECTOPIÆ.—Displacements.

| | | |
|---|---|---|
| 28. | Encephalocele | *Protrusion of the brain* |
| 29. | Omphalocele | *Umbilical hernia* |
| 30. | Bubonocele | *Inguinal hernia* |
| 31. | Oscheocele | *Scrotal hernia* |

#### Ordo VIII. ASSYMETRIA.—Anti-symmetrical conformation.

| | | |
|---|---|---|
| 32. | Cretinismus | *Cretinism* |
| 33. | Humeri dispares | *Uneven shoulders* |
| 34. | Claviculæ dispares | *Uneven collar-bones* |
| 35. | Obstipitas capitis | *Obliquity of the head* |

| | |
|---|---|
| G. 36. Gibbositas | *Humpback* |
| 37. Spina tortuosa | *Distorted spine* |
| 38. Rachitis | *Rickets* |
| 39. Claudicatio | *Lameness* |
| 40. Lordosis | *Bandy-legged* |
| 41. Scauri-peda | *Club-footed* |

Ordo IX. Paræsthesis.—Defective action of senses.

| | |
|---|---|
| 42. Cecitas | *Blindness* |
| 43. Strabismus | *Squinting* |
| 44. Surditas | *Deafness* |

Ordo X. Metrocelis.—Marks.

| | |
|---|---|
| 45. Nævi materni | *Mother's marks* |

Classis II. TOCICA.—*Congenite Diseases, dependent on Parturition.*

Ordo I. Topici.

| | | |
|---|---|---|
| G. 1. Ophthalmia | *Ophthalmy* | |
| 2. Capitis acuminatio | *Elongation of the head* | |
| 3. Integumentorum cranii tumefactio | *Tumor of the hairy scalp* | *after passive labours.* |
| 4. Contusiones et Lacerationes | *Contusions and lacerations* | |
| 5. Luxationes et Fracturæ | *Dislocations and fractures* | |

Ordo II. Universales.

| | |
|---|---|
| 6. Tarde spiritum ducere | *Retarded animation* |
| 7. Apoplexia | *Apoplexy* |
| 8. Asphyxia | *Asphyxy* |
| 9. Syphilis | *Venereal disease* |
| 10. Asthenia | *Debility* |

### Divisio Secunda.

### MORBI SUBSEQUENTES.

Classis I. ZOTICA.—*Morbid Alterations of the Preservative Functions.*

Ordo I. Pneumatici.—Affecting respiration.

| | |
|---|---|
| G. 1. Angina mitis | *Common sore throat* |
| 2. Angina gangrenosa | *Putrid sore throat* |
| 3. Angina trachealis | *Croup* |
| 4. Catarrhus | *Catarrh* |
| 5. Catarrhus suffocativus | *Spasmodic catarrh* |
| 6. Tussis simplex | *Cough* |
| 7. Pertussis | *Whooping cough* |
| 8. Pulmonea | *Inflammation of the lungs* |

Ordo II. Hæmatici.—Affecting circulation.

| | |
|---|---|
| 9. Omphalorrhœa | *Bleeding at the navel* |
| 10. Epistaxis | *Bleeding at the nose* |
| 11. Erysipelas | *St. Anthony's fire* |
| 12. Cephalitis | *Inflammation of the brain* |
| 13. Pleuritis | *Inflammation of the chest* |
| 14. Hepatitis | *Inflammation of the liver* |
| 15. Peritonitis | *Inflammation of the belly* |
| 16. Enteritis | *Inflammation of the bowels* |
| 17. Ephemera | *One-day fever* |
| 18. Variola | *Small-pox* |
| 19. Rubeola | *Measles* |
| 20. Varicella | *Chicken-pox* |
| 21. Roseola | *Rose-rash* |
| 22. Scarlatina | *Scarlet fever* |
| 23. Vaccinia | *Cow-pox* |
| 24. Urticaria | *Nettle-rash* |

**Classis II. AUXITICA.**—*Morbid Alterations of the Augmentive Functions.*

Ordo I. Cæliaci.—Affecting digestion.

| | | |
|---|---|---|
| G. 1. | Dentitio difficilis | *Painful teething* |
| 2. | Flatus | *Wind* |
| 3. | Vomitus | *Sickness of the Stomach* |
| 4. | Oxycardia | *Acidity in the Stomach* |
| 5. | Colica | *Gripes* |
| 6. | Obstipatio | *Costiveness* |
| 7. | Lienteria | *Watery gripes* |
| 8. | Diarrhœa | *Looseness* |
| 9. | Icterus | *Yellow gum* |
| 10. | Vermes | *Worms* |
| 11. | Prolapsus recti | *Falling of the gut* |

Ordo II. Lymphatici.—Affecting absorption.

| | | |
|---|---|---|
| 12. | Febris intestinalis | *Infantile remittent fever* |
| 13. | Cachexia | *Cachexy* |
| 14. | Tabes | *Mesenteric consumption* |
| 15. | Marasmus | *Decline* |
| 16. | Scrofula | *Evil* |
| 17. | Parabysma | *Swelling and hardness of the belly* |

**Classis III. APOCRITICA.**—*Morbid Alterations of the Segregating Functions.*

Ordo I. Eccritici.—Affecting secretion and excretion.

| | | |
|---|---|---|
| G. 1. | Meconii expulsio tarda | *Retention of the meconium* |
| 2. | Coriago | *Induration of the cellular membrane* |
| 3. | Coryza | *Running from the nose* |
| 4. | Ptyalismus | *Great flow of saliva* |
| 5. | Aphthæ | *Thrush* |
| 6. | Psorophthalmia | *Purulent ophthalmia* |
| 7. | Hydrencephalus | *Dropsy of the brain* |
| 8. | Hydrothorax | *Dropsy of the chest* |
| 9. | Ascites | *Dropsy of the belly* |
| 10. | Hydrocele | *Dropsy of the scrotum* |
| 11. | Paruria inops | *Scanty secretion of urine* |
| 12. | Paruria retentionis | *Stoppage of urine* |
| 13. | Paruria incontinens | *Incontinence of urine* |
| 14. | Paruria noctiflua | *Incontinence of urine during sleep* |
| 15. | Polysarcia adiposa | *Obesity* |
| 16. | Osteomalacia | *Softness of the bones* |
| 17. | Calculi | *Stone in the bladder* |

Ordo II. Dermatici.—Affecting exhalation.

| | | |
|---|---|---|
| 18. | Perspiratio vitiosa | *Defective cutaneous transudation* |
| 19. | Strophulus | *Tooth-rash* |
| 20. | Lichen | *Lichen-rash* |
| 21. | Crusta lactea | *Milky tetter* |
| 22. | Prurigo | *Itching* |
| 23. | Scabies | *Itch* |
| 24. | Intertrigo | *Chafing* |
| 25. | Impetigo | *Humid tetter* |
| 26. | Porrigo | *Scalled head* |
| 27. | Herpes | *Ring-worm* |
| 28. | Icthyosis | *Fishy scales* |
| 29. | Maculæ syphiliticæ | *Venereal blotches* |
| 30. | Psoriasis | *Scaly tetter* |
| 31. | Pityriasis | *Dandriff* |
| 32. | Purpura | *Red spots* |
| 33. | Excoriatio pone aures | *Running behind the ears* |

| | |
|---|---|
| 34. Ulceratio penis vel scroti...... | *Ulceration of the penis or scrotum* |
| 35. Erosio maxillaris............ | *Canker* |
| 36. Erosio linguæ et labiorum.... | |
| 37. Pompholigmus.................. | *Blains* |
| 38. Lepidosis..................... | *Tetter* |

Classis IV. ÆSTHETICA.—*Morbid Alterations of the Sensitive Functions.*

Ordo I. Nevrotici.—Affecting Sensation.

| | |
|---|---|
| G. 1. Singultus..................... | *Hiccough* |
| 2. Febris nervosa ............... | *Nervous fever* |
| 3. Insomnia ...................... | *Sleeplessness* |
| 4. Incubus ....................... | *Night-mare* |
| 5. Moria imbecillis ............. | *Imbecility* |
| 6. Paræsthesis .................. | *Depraved senses* |

Ordo II. Myotici.—Affecting voluntary motion.

| | |
|---|---|
| 7. Tremor......................... | *Trembling* |
| 8. Trismus nascentium .......... | *Locked-jaw* |
| 9. Convulsiones ................ | *Convulsions* |
| 10. Carus ........................ | *Stupor* |
| 11. Tetanus ..................... | *Tetanus* |
| 12. Epilepsia .................. | *Falling sickness* |
| 13. Chorea ...................... | *St. Vitus's dance* |
| 14. Loxia ....................... | *Stiff-neck* |
| 15. Contractura ................ | *Cramp* |
| 16. Nictitatio .................. | *Twinkling of the eye-lids* |
| 17. Psellismus ................. | *Stuttering* |

8, *Saville-row; October* 16, 1820.

